# Dementia is a surrogate for frailty in hip fracture mortality prediction

**DOI:** 10.1007/s00068-022-01960-9

**Published:** 2022-03-30

**Authors:** Maximilian Peter Forssten, Ioannis Ioannidis, Ahmad Mohammad Ismail, Gary Alan Bass, Tomas Borg, Yang Cao, Shahin Mohseni

**Affiliations:** 1 Department of Orthopedics Surgery, Orebro University Hospital, 701 85 Orebro, Sweden; 2 Division of Trauma and Emergency Surgery, Department of Surgery, Orebro University Hospital, 701 85 Orebro, Sweden; 3 School of Medical Sciences, Orebro University, 702 81 Orebro, Sweden; 4 Division of Traumatology, Surgical Critical Care and Emergency Surgery, University of Pennsylvania, Penn Presbyterian Medical Center, Philadelphia, PA 19104 USA

**Keywords:** Hip fracture, Dementia, Frailty, Mortality prediction, Permutation importance, Logistic regression

## Abstract

**Purpose:**

Among hip fracture patients both dementia and frailty are particularly prevalent. The aim of the current study was to determine if dementia functions as a surrogate for frailty, or if it confers additional information as a comorbidity when predicting postoperative mortality after a hip fracture.

**Methods:**

All adult patients who suffered a traumatic hip fracture in Sweden between January 1, 2008 and December 31, 2017 were considered for inclusion. Pathological fractures, non-operatively treated fractures, reoperations, and patients missing data were excluded. Logistic regression (LR) models were fitted, one including and one excluding measurements of frailty, with postoperative mortality as the response variable. The primary outcome of interest was 30-day postoperative mortality**.** The relative importance for all variables was determined using the permutation importance. New LR models were constructed using the top ten most important variables. The area under the receiver-operating characteristic curve (AUC) was used to compare the predictive ability of these models.

**Results:**

121,305 patients were included in the study. Initially, dementia was among the top ten most important variables for predicting 30-day mortality. When measurements of frailty were included, dementia was replaced in relative importance by the ability to walk alone outdoors and institutionalization. There was no significant difference in the predictive ability of the models fitted using the top ten most important variables when comparing those that included [AUC for 30-day mortality (95% CI): 0.82 (0.81–0.82)] and excluded [AUC for 30-day mortality (95% CI): 0.81 (0.80–0.81)] measurements of frailty.

**Conclusion:**

Dementia functions as a surrogate for frailty when predicting mortality up to one year after hip fracture surgery. The presence of dementia in a patient without frailty does not appreciably contribute to the prediction of postoperative mortality.

**Supplementary Information:**

The online version contains supplementary material available at 10.1007/s00068-022-01960-9.

## Introduction

As life expectancy and median population age increases globally, the incidence of hip fractures is expected to increase in the developed world [1, 2]. Hip fracture patients are a heterogenous population that on average has a significantly higher age and comorbidity burden compared to the general population [3–7]. Among these comorbidities, dementia is particularly prevalent, with up to 30% of hip fracture patients suffering from this condition [5, 7–9]. The healthcare burden associated with this patient group is accordingly expected to increase during the coming years, which makes it vital to find tools that can be used to efficiently allocate resources to the patients who need them the most [10–12].

Frailty, a reduced physiologic reserve to withstand external stressors, is common among hip fracture patients [13–15]. Frailty may in part explain the increased risk of mortality and morbidity following hip fracture surgery [13–26]. Current data indicates that up to one third of hip fracture patients die within the first postoperative year [3, 7, 27]. Furthermore, frailty has been found to have a reciprocal relationship with dementia, with it both functioning as a risk factor for the development of dementia as well as being a possible result of dementia [28–30]. Previous research has found that dementia is one of the most important variables associated with both short- and long-term mortality after hip fracture surgery [31, 32]. The aim of the current study was to determine if dementia merely functions as a surrogate for frailty, or if it confers additional information as a comorbidity when predicting postoperative mortality after a traumatic hip fracture.

## Methods

Data was obtained from the Swedish National Quality Registry for Hip Fractures, RIKSHÖFT [33]. This registry was started in 1988 with the purpose of tracking the effects of improvements in medical and surgical treatment, nursing, as well as rehabilitation on hip fracture patients in Sweden [33]. It is contributed to by nearly all orthopedic departments in Sweden, and has achieved the highest possible certification for a national quality registry in Sweden [34, 35]. All adult patients (18 or older) who suffered a primary traumatic hip fracture in Sweden between January 1, 2008 and December 31, 2017 were considered for inclusion. Pathological fractures, non-operatively treated fractures, and reoperations were excluded in order to reduce heterogeneity in the study population, along with patients with missing data. Variables retrieved from this database included age, sex, American Society of Anesthesiologists (ASA) classification, type of fracture, type of surgery, date and time of hospital admission, date and time of surgery, date of hospital discharge, and measurements of frailty such as non-independent functional status, living arrangements, walking ability, and use of walking aids [36]. This data was cross-referenced with the Swedish National Board of Health and Welfare’s Cause of Death and Patient Registers in order to obtain variables pertaining to mortality and comorbidities. The comorbidity data was used to calculate both the Charlson Comorbidity Index (CCI) and Revised Cardiac Risk Index (RCRI) [37, 38]. Data relating to the prescription of beta-blockers (ATC codes C07AA, C07AB, C07AG), which has shown an strong association with survival after hip fracture surgery, were obtained from the Swedish Prescribed Drug registry [39–42]. Patients who filled a prescription within the year before and after surgery were defined as having ongoing beta-blocker therapy [39–42]. The study was approved by the Swedish National Review Authority (ref: 2021-05403-02) and adhered to the Declaration of Helsinki [43].

## Statistical analysis

For the purpose of summarizing the cohorts, patients were divided into those that died and survived 30-days postoperatively. Furthermore, 90-day and 1-year mortality data are reported in supplementary tables. Continuous variables were presented as medians and interquartile ranges as they were non-normally distributed while categorical variables were summarized using counts and percentages. The Mann–Whitney *U*-test was used to determine the statistical significance of differences between continuous variables. The Chi-squared test or Fisher’s exact test were used as appropriate for categorical variables. The primary outcome of interest was predicting 30-day postoperative mortality, with predicting 90-day and 1-year mortality being included as secondary outcomes of interest.

Logistic regression (LR) models were constructed with 30-day, 90-day, and 1-year postoperative mortality as the response variables [31, 32]. The variables that were included as potential predictors were age, sex, ASA classification, type of surgery, type of fracture, out-of-hours surgery, time to surgery, ongoing beta-blocker therapy, CCI, RCRI, comorbidities including dementia, arrhythmia, hypertension, previous myocardial infarctions, congestive heart failure, cerebrovascular disease, chronic obstructive pulmonary disease (COPD), connective tissue diseases, diabetes mellitus, peptic ulcer disease, liver disease, hemiplegia, chronic kidney disease, local cancer, and metastatic carcinoma, as well as measurements of frailty [5, 7, 36, 39–42, 44–48]. Out-of-hours surgery was defined as surgery initiated between 17:00 and 8:00 [44]. The RCRI was treated as a continuous variable in the models while the CCI was retained as a categorical variable [7].

For each outcome two models were created. One including all variables except for the measurements of frailty and another also including the measurements of frailty. The relative importance for all variables in each model was determined using the permutation importance (PI) [49]. The PI was measured by calculating how much a predetermined value [1- Area under the receiver-operating characteristic curve (AUC)] was affected by the omission of a specific variable. Rather than simply eliminating the variable from the dataset, this method replaces it with noise from other cases by rearranging the values of the variable in order to mask the information of a variable during evaluation. To account for the inherent uncertainty related to the use of permutations, this process was repeated 10 times for each model. The relative importance of each variable in the model was then presented as the average increase in 1-AUC relative to the AUC in a model including all variables without masking, which means that the value for an individual variable was equivalent to the percentage improvement in the AUC from including that variable.

Finally, 6 new LR models were constructed using the top ten most important variables determined by their PI [31, 32]. The AUC for each model was calculated and used to compare the predictive ability of the two models, one with and one without measurements of frailty, for each outcome. This was done in order to determine if the measurements of frailty improved the predictive ability of the models, or if they functioned as a replacement for dementia.

The models with 90-day mortality as the response variable excluded patients who died within the first 30 days after surgery while the models predicting 1-year postoperative mortality excluded patients who had died within 90 days. This allowed for the identification of the variables with the greatest predictive ability for each timepoint in isolation. This is particularly important when predicting 1-year mortality as this reduces the effect of early deaths due to postoperative complications as well as the higher prevalence of advanced directives present in the study population compared to the general population [32, 40].

Statistical significance was defined as a two-sided p-value < 0.05. Analyses were performed using the statistical programming language R (R Foundation for Statistical Computing, Vienna, Austria) using the Tidyverse, DALEX, Haven, Lubridate, Cowplot, and Parallel packages [50].

## Results

A total of 121,305 patients were included in the current study. Patients who died within 30 days postoperatively tended to be older (88 years vs 83 years, *p* < 0.001), more often male (45.9% vs 30.8%, *p* < 0.001), and less fit for surgery according to their ASA classification (ASA ≥ 3: 81.2% vs 56.3%, *p* < 0.001). They were also less likely to have ongoing beta-blocker therapy (19.5% vs 40.7%, *p* < 0.001), or have been operated within 24 h (64.9% vs 69.2%, *p* < 0.001); however, there was no clinically significant difference in the proportion operated out-of-hours (30.7% vs 29.6%, *p* = 0.033). Extracapsular fractures were slightly more common among patients who died (48.3% vs 45.9%, *p* < 0.001), while the type of surgery did not differ significantly (Internal fixation: 67.3% vs 66.7%, *p* = 0.258). Patients who died also had a higher comorbidity burden according to both the RCRI (RCRI ≥ 2: 27.2% vs 12.4%, *p* < 0.001) and CCI (CCI ≥ 7: 36.8% vs 17.3%, *p* < 0.001). Accordingly, almost all comorbidities were more prevalent among patients who died within 30 days, except for hypertension and hemiplegia which were more prevalent among patients who survived, and connective tissue disease which was equally prevalent in both cohorts. These patients also tended to be frailer with a higher proportion being institutionalized (48.4% vs 22.9%, *p* < 0.001), being unable to walk outside alone (67.7% vs 38.6%, *p* < 0.001), and requiring a walker, wheelchair or being bedridden prior to surgery (69.3% vs 48.1%, *p* < 0.001) (Table 1). These trends remained relatively unchanged when comparing patients who lived and died 90 days and 1 year postoperatively. Differences in trends at the different timepoints were mainly seen in the prevalence of beta-blocker therapy, type of surgery, and the previously mentioned comorbidities (Supplemental Tables 1 and 2).

**Table 1:** Demographics and clinical characteristics of hip fracture patients who were alive and dead 30 days postoperatively

	Alive (*N* = 112,413)	Dead (*N* = 8892)	*P* value
Age, median [IQR]	83 [76–88]	88 [83–92]	< 0.001
Sex, *n* (%)			
Female	77,767 (69.2)	4815 (54.1)	< 0.001
Male	34,646 (30.8)	4077 (45.9)	
Ongoing beta-blocker therapy, n (%)	45,709 (40.7)	1733 (19.5)	< 0.001
ASA classification, *n* (%)			
1	6064 (5.4)	107 (1.2)	< 0.001
2	42,994 (38.2)	1562 (17.6)	
3	55,699 (49.5)	5343 (60.1)	
4	7587 (6.7)	1835 (20.6)	
5	69 (0.1)	45 (0.5)	
Time to surgery, *n* (%)			
< 12 h	25,878 (23.0)	2017 (22.7)	< 0.001
12–24 h	51,990 (46.2)	3753 (42.2)	
24–36 h	19,715 (17.5)	1783 (20.1)	
36–48 h	8464 (7.5)	697 (7.8)	
≥ 48 h	6366 (5.7)	642 (7.2)	
Out-of-hours surgery, *n* (%)	33,315 (29.6)	2731 (30.7)	0.033
Type of fracture, *n* (%)			
Intracapsular fracture	60,760 (54.1)	4597 (51.7)	< 0.001
Extracapsular fracture	51,653 (45.9)	4295 (48.3)	
Type of surgery, *n* (%)			
Internal fixation	74,958 (66.7)	5982 (67.3)	0.258
Arthroplasty	37,455 (33.3)	2910 (32.7)	
RCRI, *n* (%)			
0	68,069 (60.6)	3534 (39.7)	< 0.001
1	30,353 (27.0)	2934 (33.0)	
2	10,268 (9.1)	1574 (17.7)	
3	2968 (2.6)	650 (7.3)	
4	655 (0.6)	178 (2.0)	
5	100 (0.1)	22 (0.2)	
Charlson comorbidity index, *n* (%)			
≤ 4	51,871 (46.1)	1705 (19.2)	< 0.001
5–6	41,091 (36.6)	3919 (44.1)	
≥ 7	19,451 (17.3)	3268 (36.8)	
Arrhythmia, *n* (%)	20,399 (18.1)	2391 (26.9)	< 0.001
Hypertension, *n* (%)	44,127 (39.3)	3362 (37.8)	0.007
Previous myocardial infarction, *n* (%)	6162 (5.5)	1125 (12.7)	< 0.001
Congestive heart failure, *n* (%)	15,798 (14.1)	3140 (35.3)	< 0.001
Cerebrovascular disease, *n* (%)	19,142 (17.0)	2064 (23.2)	< 0.001
COPD, *n* (%)	12,638 (11.2)	1451 (16.3)	< 0.001
Connective tissue disease, *n* (%)	5522 (4.9)	404 (4.5)	0.127
Peptic ulcer disease, *n* (%)	3604 (3.2)	360 (4.0)	< 0.001
Dementia, *n* (%)	20,975 (18.7)	3049 (34.3)	< 0.001
Diabetes, *n* (%)	16,551 (14.7)	1495 (16.8)	< 0.001
Liver disease, *n* (%)	1121 (1.0)	125 (1.4)	< 0.001
Hemiplegia, *n* (%)	2529 (2.2)	167 (1.9)	0.024
Chronic kidney disease, *n* (%)	5260 (4.7)	1030 (11.6)	< 0.001
Local cancer, *n* (%)	11,994 (10.7)	1278 (14.4)	< 0.001
Metastatic carcinoma, *n* (%)	2286 (2.0)	406 (4.6)	< 0.001
Non-independent functional status, *n* (%)	56,937 (50.6)	6395 (71.9)	< 0.001
Living arrangements, *n* (%)			
Living alone	52,454 (46.7)	2864 (32.2)	< 0.001
Not living alone	34,169 (30.4)	1725 (19.4)	
Institutionalized	25,790 (22.9)	4303 (48.4)	
Walking ability, *n* (%)			
Walk alone outdoors	69,011 (61.4)	2872 (32.3)	< 0.001
Walk with company outdoors	9567 (8.5)	1123 (12.6)	
Walk alone indoors	23,521 (20.9)	3235 (36.4)	
Walk with company indoors	7321 (6.5)	1203 (13.5)	
Unable to walk	2993 (2.7)	459 (5.2)	
Walking aid, *n* (%)			
No walking aid	49,190 (43.8)	2134 (24.0)	< 0.001
One walking aid	6984 (6.2)	462 (5.2)	
Two walking aids	2133 (1.9)	140 (1.6)	
Walker	50,361 (44.8)	5573 (62.7)	
Wheelchair or bedridden	3745 (3.3)	583 (6.6)	

*ASA* American society of anesthesiologists, *RCRI* revised cardiac risk index, *COPD* chronic obstructive pulmonary disease

**Fig. 1:**
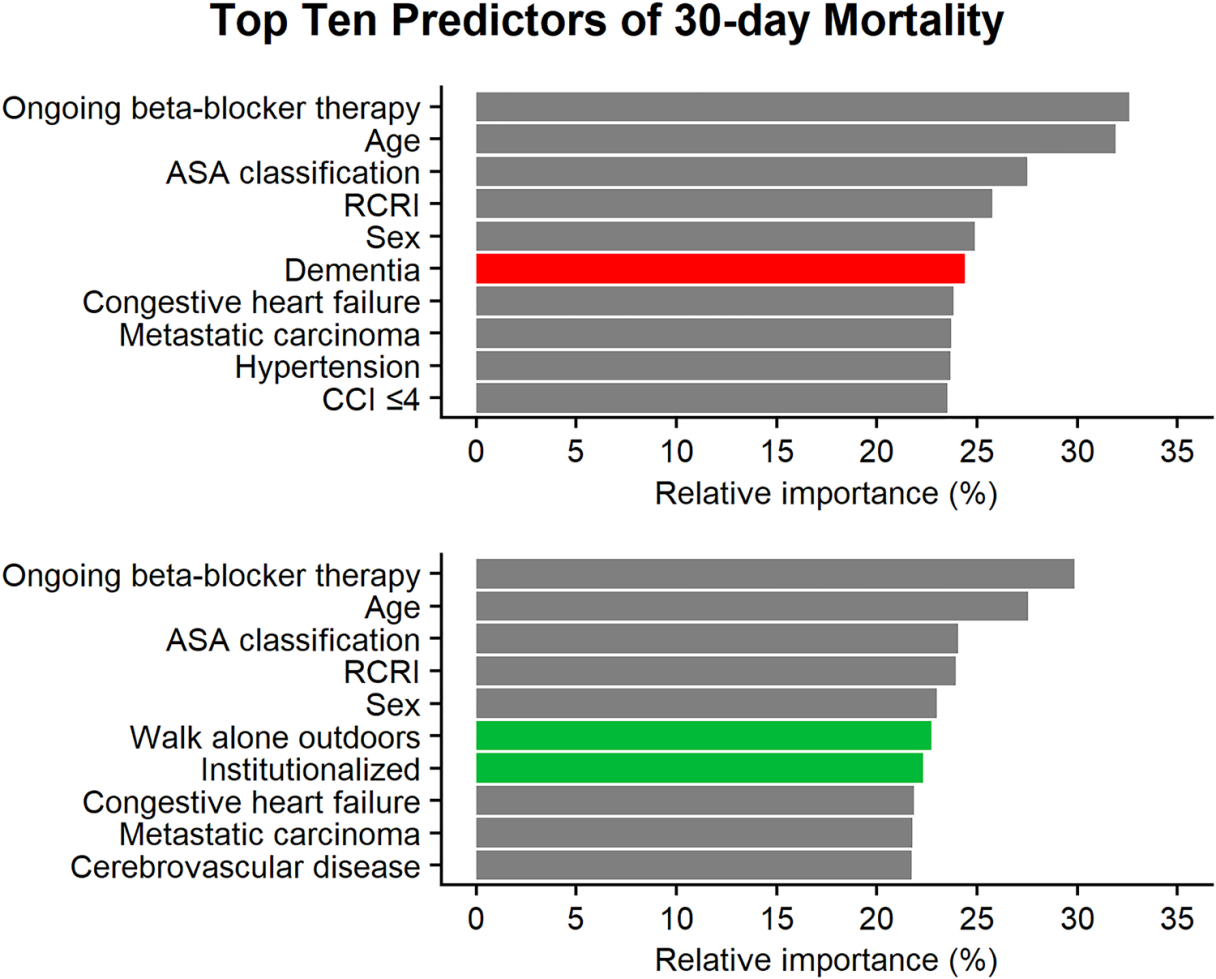
Top ten predictors of 30-day postoperative mortality with a logistic regression model. In the upper plot measurements of frailty were excluded when calculating the relative importance while the lower plot used all variables (including both dementia and measurements of frailty). *ASA* American society of anesthesiologists, *CCI* Charlson comorbidity index, *RCRI* revised cardiac risk index

When ranking the relative importance of variables for predicting 30-day postoperative mortality, excluding measurements of frailty, the top ten variables were ongoing beta-blocker therapy, age, ASA classification, RCRI, sex, dementia, congestive heart failure, metastatic carcinoma, hypertension, and CCI ≤ 4. When measurements of frailty were included the top ten variables were instead ongoing beta-blocker therapy, age, ASA classification, RCRI, sex, the ability to walk alone outdoors, institutionalization, congestive heart failure, metastatic carcinoma, and cerebrovascular disease (Fig. 1).

**Fig. 2:**
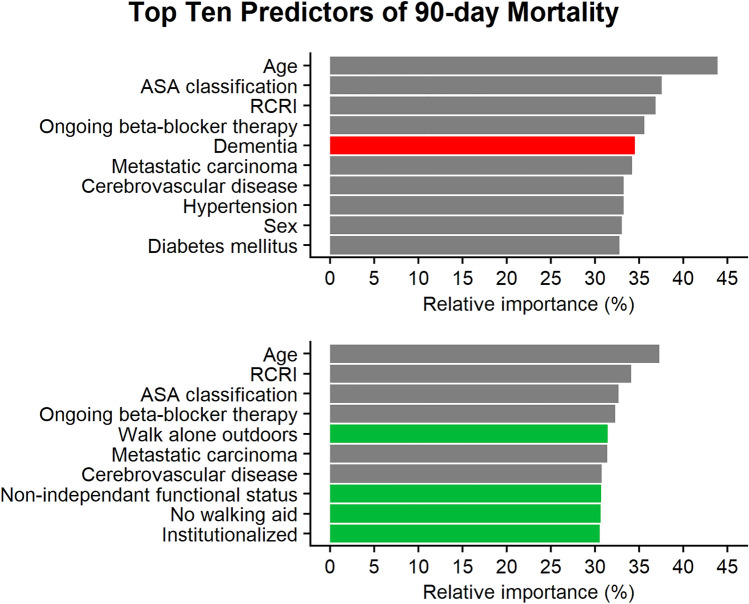
Top ten predictors of 90-day postoperative mortality with a logistic regression model. In the upper plot measurements of frailty were excluded when calculating the relative importance while the lower plot used all variables (including both dementia and measurements of frailty). *ASA* American society of anesthesiologists, *RCRI* revised cardiac risk index

For predicting 90-day mortality, excluding measurements of frailty, the top ten variables were age, ASA classification, RCRI, ongoing beta-blocker therapy, dementia, metastatic carcinoma, cerebrovascular disease, hypertension, sex, and diabetes mellitus. With the inclusion of measurements of frailty, the top ten variables were instead age, RCRI, ASA classification, ongoing beta-blocker therapy, the ability to walk alone outdoors, metastatic carcinoma, cerebrovascular disease, non-independent functional status, the ability to walk without a walking aid, and institutionalization (Fig. 2).

**Fig. 3:**
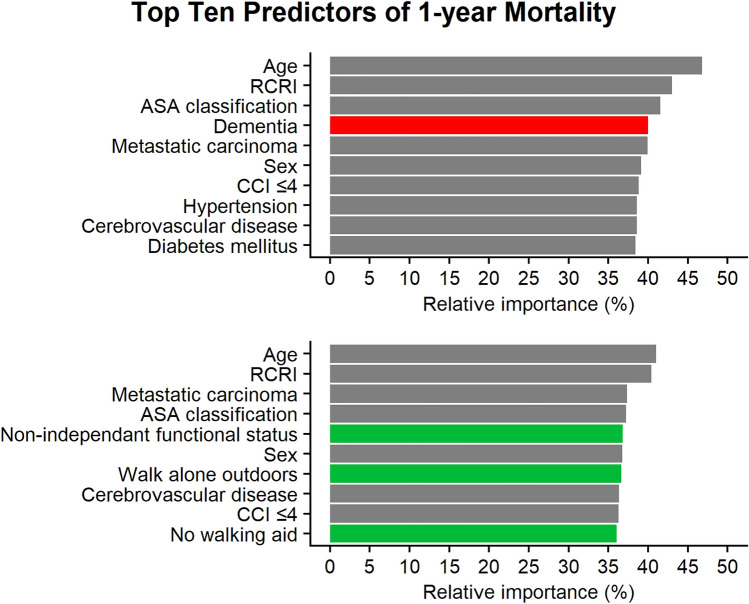
Top ten predictors of 1-year postoperative mortality with a logistic regression model. In the upper plot measurements of frailty were excluded when calculating the relative importance while the lower plot used all variables (including both dementia and measurements of frailty). *ASA* American society of anesthesiologists, *CCI* Charlson comorbidity index, *RCRI* revised cardiac risk index

For 1-year mortality, the top ten variables for prediction after excluding measurements of frailty were age, RCRI, ASA classification, dementia, metastatic carcinoma, sex, CCI ≤ 4, hypertension, cerebrovascular disease, and diabetes mellitus. With measurements of frailty, the top ten variables instead included age, RCRI, metastatic carcinoma, ASA classification, non-independent functional status, sex, the ability to walk alone outdoors, cerebrovascular disease, CCI ≤ 4, diabetes mellitus, and the ability to walk without a walking aid (Fig. 3).

In regard to 30-day, 90-day, and 1-year mortality, there was no significant difference in the predictive ability of the LR models built using the top ten most important variables when comparing those that included and excluded measurements of frailty. When all 41 variables were used to build the LR models, the predictive ability of the model remained relatively unchanged for 30-day postoperative mortality, while improvements were seen for 90-day [AUC (95% CI): 0.81 (0.80–0.81)] and 1-year postoperative mortality [AUC (95% CI): 0.78 (0.78–0.79)] (Table 2).

**Table 2:** Predictive ability of logistic regression models built using the top ten most important variables, as well as all the variables available in the dataset

Outcome	AUC for models excluding frailty (95% CI)	AUC for models including frailty (95% CI)	AUC for models using all variables (95% CI)
30-day mortality	0.81 (0.80–0.81)	0.82 (0.81–0.82)	0.82 (0.82–0.83)
90-day mortality	0.75 (0.75–0.76)	0.76 (0.75–0.76)	0.81 (0.80–0.81)
1-year mortality	0.72 (0.71–0.72)	0.72 (0.72–0.73)	0.78 (0.78–0.79)

*AUC* area under the receiver operating characteristic curve, *CI* confidence interval

## Discussion

This is the first study to investigate the contribution of dementia in predicting postoperative mortality in hip fracture patients. The results demonstrate that when measurements of frailty are included in the predictive models, dementia loses its predictive ability and is instead replaced by measurements of frailty while the remaining variables’ relative importance are relatively unchanged. When predicting 30-day mortality, dementia is replaced by the ability to walk without a walking aid, and institutionalization. For 90-day mortality, dementia is replaced in importance by the ability to walk alone outdoors, non-independent functional status, the ability to walk without a walking aid, and institutionalization. Finally, for 1-year mortality, non-independent functional status, the ability to walk alone outdoors, and the ability to walk without a walking aid takes precedence over dementia. This interchangeability is further bolstered by the fact that the predictive ability of the LR models were virtually identical when dementia or measurements of frailty were used.

Frailty can be defined as a condition characterized by a reduced physiologic reserve to withstand stressors that is coupled with an increased susceptibility to morbidity, disability, and mortality as a result of a degeneration in multiple organ systems [17–26]. In regard to hip fractures, frail patients with dementia have been found to be less likely to receive in-hospital rehabilitation, while frail patients that do receive rehabilitation often experience poorer outcomes [51, 52]. Frailty has also been observed to be associated with increased mortality, morbidity, as well as a reduced quality of life after hip fracture surgery [53–55]. Moreover, frail patients are at an increased risk of developing dementia, which may partially explain the high prevalence of dementia among hip fracture patients [9, 29, 56]. While no previous studies have directly compared the predictive ability of frailty and dementia, prior analyses have demonstrated that dementia is one of the most important predictors of postoperative mortality in hip fracture patients [31, 32]. However, measurements of frailty were not included in these analyses [31, 32].

The results of the current analyses suggest that, in the inpatient setting, dementia indicates the presence of other hallmarks of frailty. This could both include the measurements of frailty available in our own database, such as non-independent functional status, institutionalization, the inability to walk alone outside, as well as the need for a walking aid, along with those traditionally cited when discussing frailty as a syndrome, including unintentional weight loss, self-reported exhaustion, weakness, slow walking speed, and low physical activity [20]. The presence of many of these hallmarks may signify the need for additional interventions, such as further preoperative optimization and specialized orthogeriatric care [57, 58]. The presence of these hallmarks may also play an important role in helping physicians efficiently and equitably allocate health care resources, such as access to tools and personal required for postoperative rehabilitation. In the context of mortality prediction, these results indicate that these measurements of frailty can be replaced by dementia when better alternatives, such as more detailed frailty indices, are unavailable or unfeasible to calculate [36]. Furthermore, while dementia is often discussed as a discrete condition, the reality is that dementia is the result of several different conditions with varying pathophysiology and mortality rates [59, 60]. However, since dementia only serves as an indicator of frailty this distinction is not as important when *predicting* mortality. Consequently, concentrating on a broader range of variables might yield more useful results than focusing on the granular details of each patient’s dementia when gathering data for the purpose of predicting postoperative mortality in hip fracture patients.

It has often been stated that dementia is a cause of mortality [60–64]. However, this study provides further evidence for why this line of reasoning is incorrect. Previous studies have demonstrated that dementia is a risk factor for mortality in hip fracture patients [65–67]. It has also been found that these patients die from the same causes as all other hip fracture patients, but to a much greater extent [8]. The most common causes of death appear to be cardiovascular events and multiorgan failure, corresponding to trends observed in the general hip fracture population [8]. Nonetheless, the mechanism behind the higher mortality rate observed among hip fracture patients with dementia, compared to the general hip fracture population, remains an open question. The results of this study suggest that it may be due to frailty. Patients are not dying due to the dementia itself, but rather the higher degree of frailty associated with it. Consequently, when these patients are exposed to the stress of the initial hip fracture and subsequent surgery, their physiologic reserve is severely reduced, thereby limiting their ability to maintain homeostasis [16, 42].

While the exact measurements of frailty vary slightly between timepoints, the ability to walk alone is always included as one of the top ten most important predictors of both short-term and long-term mortality. This is conducive with previous research that demonstrates that lower preinjury mobility is associated with an elevated risk of postoperative mortality [68–71]. Early mobilization postoperatively has been identified as key to reducing hospital-related functional decline in order to reduce mortality and morbidity [71–73]. As it stands, a significant proportion of hip fracture patients are unable to regain their pre-facture level of mobility [74, 75]. Achieving early mobilization can therefore already be challenging in this patient population. If the patient also has a reduced walking ability at the onset of this process, this merely serves to further increase the difficulty of achieving full mobilization. Accordingly, the inability to walk alone outdoors prior to injury will increase the risk of immobility and consequently also elevate the risk of poorer functional outcomes, complications such as pressure ulcers, thromboembolism, and pneumonia, as well as mortality [76–78].

While this study was able to make use of an extensive national database based on 10 years of consecutive hip fracture patients, there are also limitations that bear discussing. Foremost among these is understanding how to interpret the results of the current study. The results of the analyses indicate that dementia functions as a surrogate for frailty when *predicting* mortality in hip fracture patients. This does not necessarily mean that all patients with dementia are frail. Nevertheless, what these results do demonstrate is that there is likely a strong correlation between dementia and frailty. If not, then dementia could not function as a surrogate. The results also show that the presence of dementia in a patient *without* frailty does not appear to contribute meaningfully to the prediction of mortality after hip fracture surgery. This study also assumes that walking ability, walking aids, living arrangements, and functional status are markers of frailty; while this is based on the work and validations performed by previous researchers it is important to understand that these variables merely indicate the presence of frailty rather than embodying frailty itself [36, 79–81]. Finally, the regular caveats that apply to retrospective studies are present. The analyses were limited to the variables available in the database, which is why additional measures of frailty were not included. The predictive importance of intraoperative variables and anesthesiologic considerations could also not be evaluated; however, their inclusion would likely only have shifted ranks of the individual variables without changing the conclusions, since dementia is clearly replaced by markers of frailty in relative importance at all timepoints. The study is also reliant on the validity of the RIKSHÖFT database. Fortunately, it is a highly regarded database, with a case coverage between 80–90%, that is contributed to by the majority of orthopedic departments in Sweden [82, 83]. The risk of selection bias is relatively low given that RIKSHÖFT is intended to capture all hip fracture patients in Sweden; nevertheless, it should be considered given the observational nature of the data. The large sample size also reduces the effect of any non-differential misclassification, which would otherwise bias the results towards the null. Differential misclassification is more challenging to manage and should always be considered when comparing patients with and without dementia. The current analysis consequently only used variables which could be objectively verified independently of the patients’ cognitive ability.

## Conclusion

For the purpose of predicting mortality up to one year after hip fracture surgery, dementia functions as a surrogate for frailty in this patient population. On the other hand, the presence of dementia in a patient without frailty does not appreciably contribute to the prediction of postoperative mortality. Dementia may also be used as a replacement for frailty when predicting postoperative mortality in hip fracture patients when better alternatives are unavailable. Additional research is indicated to further investigate the distribution of frailty among hip fracture patients with dementia.

## Supplementary Information

Below is the link to the electronic supplementary material.

Supplementary file1 (DOCX 26 KB)
