# Supplementary material for: Dementia is a surrogate for frailty in hip fracture mortality prediction
Source: Eur J Trauma Emerg Surg. 2022 Mar 30;48(5):4157–67. doi: 10.1007/s00068-022-01960-9 (PMC9532301; doi:10.1007/s00068-022-01960-9)
Supplement: Supplementary file 1 — Supplementary file1 (DOCX 26 KB) [file 68_2022_1960_MOESM1_ESM.docx]

| **Supplemental Table 1.** Demographics and clinical characteristics of hip fracture patients who were alive and dead 90 days postoperatively, excluding those who died within 30 days | | | |
| --- | --- | --- | --- |
|  | **Alive (N = 105,650)** | **Dead (N = 6,763)** | **P-value** |
| Age, median [IQR] | 83 [76-88] | 87 [82-91] | <0.001 |
| Sex, n (%) |  |  | <0.001 |
| Female | 73,489 (69.6) | 4,278 (63.3) |  |
| Male | 32,161 (30.4) | 2,485 (36.7) |  |
| Ongoing beta-blocker therapy, n (%) | 43,542 (41.2) | 2,167 (32.0) | <0.001 |
| ASA classification, n (%) |  |  | <0.001 |
| 1 | 5,982 (5.7) | 82 (1.2) |  |
| 2 | 41,545 (39.3) | 1,449 (21.4) |  |
| 3 | 51,527 (48.8) | 4,172 (61.7) |  |
| 4 | 6,540 (6.2) | 1,047 (15.5) |  |
| 5 | 56 (0.1) | 13 (0.2) |  |
| Time to surgery, n (%) |  |  | <0.001 |
| <12 hours | 24,324 (23.0) | 1,554 (23.0) |  |
| 12-24 hours | 49,061 (46.4) | 2,929 (43.3) |  |
| 24-36 hours | 18,383 (17.4) | 1,332 (19.7) |  |
| 36-48 hours | 7,946 (7.5) | 518 (7.7) |  |
| ≥ 48 hours | 5,936 (5.6) | 430 (6.4) |  |
| Out-of-hours surgery, n (%) | 31,255 (29.6) | 2,060 (30.5) | 0.129 |
| Type of fracture, n (%) |  |  | <0.001 |
| Intracapsular fracture | 57,305 (54.2) | 3,455 (51.1) |  |
| Extracapsular fracture | 48,345 (45.8) | 3,308 (48.9) |  |
| Type of surgery, n (%) |  |  | <0.001 |
| Internal fixation | 70,177 (66.4) | 4,781 (70.7) |  |
| Arthroplasty | 35,473 (33.6) | 1,982 (29.3) |  |
| RCRI, n (%) |  |  | <0.001 |
| 0 | 64,774 (61.3) | 3,295 (48.7) |  |
| 1 | 28,245 (26.7) | 2,108 (31.2) |  |
| 2 | 9,344 (8.8) | 924 (13.7) |  |
| 3 | 2,626 (2.5) | 342 (5.1) |  |
| 4 | 582 (0.6) | 73 (1.1) |  |
| 5 | 79 (0.1) | 21 (0.3) |  |
| Charlson Comorbidity Index, n (%) |  |  | <0.001 |
| ≤4 | 50,267 (47.6) | 1,604 (23.7) |  |
| 5-6 | 38,054 (36.0) | 3,037 (44.9) |  |
| ≥7 | 17,329 (16.4) | 2,122 (31.4) |  |
| Arrhythmia, n (%) | 18,750 (17.7) | 1,649 (24.4) | <0.001 |
| Hypertension, n (%) | 41,604 (39.4) | 2,523 (37.3) | <0.001 |
| Previous myocardial infarction, n (%) | 5,610 (5.3) | 552 (8.2) | <0.001 |
| Congestive heart failure, n (%) | 14,063 (13.3) | 1,735 (25.7) | <0.001 |
| Cerebrovascular disease, n (%) | 17,778 (16.8) | 1,364 (20.2) | <0.001 |
| COPD, n (%) | 11,754 (11.1) | 884 (13.1) | <0.001 |
| Connective tissue disease, n (%) | 5,198 (4.9) | 324 (4.8) | 0.654 |
| Peptic ulcer disease, n (%) | 3,323 (3.1) | 281 (4.2) | <0.001 |
| Dementia, n (%) | 18,716 (17.7) | 2,259 (33.4) | <0.001 |
| Diabetes, n (%) | 15,436 (14.6) | 1,115 (16.5) | <0.001 |
| Liver disease, n (%) | 1,046 (1.0) | 75 (1.1) | 0.373 |
| Hemiplegia, n (%) | 2,416 (2.3) | 113 (1.7) | 0.001 |
| Chronic kidney disease, n (%) | 4,647 (4.4) | 613 (9.1) | <0.001 |
| Local cancer, n (%) | 11,057 (10.5) | 937 (13.9) | <0.001 |
| Metastatic carcinoma, n (%) | 1,879 (1.8) | 407 (6.0) | <0.001 |
| Non-independent functional status, n (%) | 52,209 (49.4) | 4,728 (69.9) | <0.001 |
| Living arrangements, n (%) |  |  | <0.001 |
| Living alone | 50,056 (47.4) | 2,398 (35.5) |  |
| Not living alone | 32,810 (31.1) | 1,359 (20.1) |  |
| Institutionalized | 22,784 (21.6) | 3,006 (44.4) |  |
| Walking ability, n (%) |  |  | <0.001 |
| Walk alone outdoors | 66,646 (63.1) | 2,365 (35.0) |  |
| Walk with company outdoors | 8,698 (8.2) | 869 (12.8) |  |
| Walk alone indoors | 21,097 (20.0) | 2,424 (35.8) |  |
| Walk with company indoors | 6,496 (6.1) | 825 (12.2) |  |
| Unable to walk | 2,713 (2.6) | 280 (4.1) |  |
| Walking aid, n (%) |  |  | <0.001 |
| No walking aid | 47,438 (44.9) | 1,752 (25.9) |  |
| One walking aid | 6,621 (6.3) | 363 (5.4) |  |
| Two walking aids | 2,030 (1.9) | 103 (1.5) |  |
| Walker | 46,164 (43.7) | 4,197 (62.1) |  |
| Wheelchair or bedridden | 3,397 (3.2) | 348 (5.1) |  |
| *ASA, American Society of Anesthesiologists; RCRI, Revised Cardiac Risk Index; COPD, chronic obstructive pulmonary disease* | | | |

| **Supplemental Table 2.** Demographics and clinical characteristics of hip fracture patients who were alive and dead 1 year postoperatively, excluding those who died within 90 days | | | |
| --- | --- | --- | --- |
|  | **Alive (N = 93,767)** | **Dead (N = 11,883)** | **P-value** |
| Age, median [IQR] | 83 [75-88] | 86 [81-91] | <0.001 |
| Sex, n (%) |  |  | <0.001 |
| Female | 66,057 (70.4) | 7,432 (62.5) |  |
| Male | 27,710 (29.6) | 4,451 (37.5) |  |
| Ongoing beta-blocker therapy, n (%) | 38,357 (40.9) | 5,185 (43.6) | <0.001 |
| ASA classification, n (%) |  |  | <0.001 |
| 1 | 5,782 (6.2) | 200 (1.7) |  |
| 2 | 38,572 (41.1) | 2,973 (25.0) |  |
| 3 | 44,328 (47.3) | 7,199 (60.6) |  |
| 4 | 5,046 (5.4) | 1,494 (12.6) |  |
| 5 | 39 (0.0) | 17 (0.1) |  |
| Time to surgery, n (%) |  |  | <0.001 |
| <12 hours | 21,654 (23.1) | 2,670 (22.5) |  |
| 12-24 hours | 43,750 (46.7) | 5,311 (44.7) |  |
| 24-36 hours | 16,152 (17.2) | 2,231 (18.8) |  |
| 36-48 hours | 7,034 (7.5) | 912 (7.7) |  |
| ≥ 48 hours | 5,177 (5.5) | 759 (6.4) |  |
| Out-of-hours surgery, n (%) | 27,597 (29.4) | 3,658 (30.8) | 0.002 |
| Type of fracture, n (%) |  |  | <0.001 |
| Intracapsular fracture | 51,073 (54.5) | 6,232 (52.4) |  |
| Extracapsular fracture | 42,694 (45.5) | 5,651 (47.6) |  |
| Type of surgery, n (%) |  |  | <0.001 |
| Internal fixation | 61,944 (66.1) | 8,233 (69.3) |  |
| Arthroplasty | 31,823 (33.9) | 3,650 (30.7) |  |
| RCRI, n (%) |  |  | <0.001 |
| 0 | 58,991 (62.9) | 5,783 (48.7) |  |
| 1 | 24,674 (26.3) | 3,571 (30.1) |  |
| 2 | 7,632 (8.1) | 1,712 (14.4) |  |
| 3 | 1,994 (2.1) | 632 (5.3) |  |
| 4 | 422 (0.5) | 160 (1.3) |  |
| 5 | 54 (0.1) | 25 (0.2) |  |
| Charlson Comorbidity Index, n (%) |  |  | <0.001 |
| ≤4 | 47,235 (50.4) | 3,032 (25.5) |  |
| 5-6 | 33,008 (35.2) | 5,046 (42.5) |  |
| ≥7 | 13,524 (14.4) | 3,805 (32.0) |  |
| Arrhythmia, n (%) | 15,813 (16.9) | 2,937 (24.7) | <0.001 |
| Hypertension, n (%) | 36,736 (39.2) | 4,868 (41.0) | <0.001 |
| Previous myocardial infarction, n (%) | 4,602 (4.9) | 1,008 (8.5) | <0.001 |
| Congestive heart failure, n (%) | 11,136 (11.9) | 2,927 (24.6) | <0.001 |
| Cerebrovascular disease, n (%) | 15,209 (16.2) | 2,569 (21.6) | <0.001 |
| COPD, n (%) | 10,103 (10.8) | 1,651 (13.9) | <0.001 |
| Connective tissue disease, n (%) | 4,589 (4.9) | 609 (5.1) | 0.283 |
| Peptic ulcer disease, n (%) | 2,799 (3.0) | 524 (4.4) | <0.001 |
| Dementia, n (%) | 15,243 (16.3) | 3,473 (29.2) | <0.001 |
| Diabetes, n (%) | 13,340 (14.2) | 2,096 (17.6) | <0.001 |
| Liver disease, n (%) | 896 (1.0) | 150 (1.3) | 0.002 |
| Hemiplegia, n (%) | 2,147 (2.3) | 269 (2.3) | 0.884 |
| Chronic kidney disease, n (%) | 3,591 (3.8) | 1,056 (8.9) | <0.001 |
| Local cancer, n (%) | 9,233 (9.8) | 1,824 (15.3) | <0.001 |
| Metastatic carcinoma, n (%) | 1,249 (1.3) | 630 (5.3) | <0.001 |
| Non-independent functional status, n (%) | 44,223 (47.2) | 7,986 (67.2) | <0.001 |
| Living arrangements, n (%) |  |  | <0.001 |
| Living alone | 45,323 (48.3) | 4,733 (39.8) |  |
| Not living alone | 30,072 (32.1) | 2,738 (23.0) |  |
| Institutionalized | 18,372 (19.6) | 4,412 (37.1) |  |
| Walking ability, n (%) |  |  | <0.001 |
| Walk alone outdoors | 61,503 (65.6) | 5,143 (43.3) |  |
| Walk with company outdoors | 7,363 (7.9) | 1,335 (11.2) |  |
| Walk alone indoors | 17,508 (18.7) | 3,589 (30.2) |  |
| Walk with company indoors | 5,199 (5.5) | 1,297 (10.9) |  |
| Unable to walk | 2,194 (2.3) | 519 (4.4) |  |
| Walking aid, n (%) |  |  | <0.001 |
| No walking aid | 44,096 (47.0) | 3,342 (28.1) |  |
| One walking aid | 5,950 (6.3) | 671 (5.6) |  |
| Two walking aids | 1,821 (1.9) | 209 (1.8) |  |
| Walker | 39,156 (41.8) | 7,008 (59.0) |  |
| Wheelchair or bedridden | 2,744 (2.9) | 653 (5.5) |  |
| *ASA, American Society of Anesthesiologists; RCRI, Revised Cardiac Risk Index; COPD, chronic obstructive pulmonary disease* | | | |
